# Pre-lacteal feeding practice and associated factors among mothers having children aged less than six months in Dilla town, Southern Ethiopia

**DOI:** 10.1186/s12887-024-04660-y

**Published:** 2024-03-23

**Authors:** Anteneh Gashaw, Haymanot Mitku

**Affiliations:** Department of Midwifery, College of Medicine & Health Sciences, Dilla University, Dilla, Ethiopia

**Keywords:** Dilla town, Ethiopia, Pre-lacteal feeding practice

## Abstract

**Background:**

Pre-lacteal feeding, the introduction of liquids or non-breast milk foods before establishing regular breastfeeding, poses significant risks to newborns, depriving them of vital nutrients and the protective benefits of colostrum while exposing them to infection hazards. Despite breast milk being a renewable and comprehensive source of infant nutrition for the first six months of life, prevalent in many low income country are pre-lacteal feeds such as honey, sugar-water, jiggery water, castor oil, and goat’s milk. These practices, widespread in such regions, carry potential risks of infection and aspiration. The objective of this study is to assess the prevalence of pre-lacteal feeding and identify associated factors among mothers with children under six months in Dilla Town, Southern Ethiopia.

**Method:**

A community-based cross-sectional study took place in Dilla town, southern Ethiopia, spanning from June 20 to August 20, 2022. The study included a total of 372 participants, selected through simple random sampling for kebele and systematic random sampling for individual participants. Data was collected using interviewer-administered structured questionnaires and subsequently coded, entered, cleaned, and edited using SPSS version 23.0 software. The presentation of data utilized tables and figures, followed by a logistic regression analysis to identify potential factors associated with pre-lacteal feeding. The significance level was set at a *p*-value less than 0.05 for the final model.

**Result:**

The prevalence of pre-lacteal feeding practice was 176 (47.3%) in the study area and having no maternal education (AOR = 3.68, 95% CI; [1.01–5.84] colostrum avoidance (AOR = 4.20, 95% CI; [2.03–6.86] and lack of breast feeding counseling (AOR = 2.00, 95% CI; 1.40–2.57), were factors associated with pre-lacteal feeding practices.

**Conclusion and recommendation:**

Pre-lacteal feeding practice among mothers of children aged less than 6 months in Dilla town was found to be higher than the national prevalence. No formal education, colostrum avoidance, lack of breastfeeding counseling, were factors associated with pre-lacteal feeding practices. So awareness creation activities on the risks of PLF (pre-lacteal feeding) and improving breastfeeding counseling targeted to all mothers and care givers including their families within the study area is vital.

## Background

Pre-lacteal feeding, defined as the introduction of liquids or non-breast milk foods before the commencement of regular breastfeeding, poses significant risks to infants. This practice not only denies the child crucial nutrients and the protective benefits of colostrum but also exposes the infant to the potential threat of illness [1]. Furthermore, the administration of pre-lacteal fluids, often done with a finger or spoon while the child is asleep or crying, carries the risk of aspiration into the airways and lungs. Consequently, this feeding method diminishes the exclusive breastfeeding practice, which can pose serious risks, even proving fatal for the infant [2, 3].

Pre-lacteal feeds are often administered with the expectation that they will serve various purposes, such as acting as laxatives, cleansers, hydrators, or aiding in the elimination of meconium [4]. Contrary to UNICEF’s recommendations advocating for immediate breastfeeding initiation after birth, pre-lacteal feeding is a common practice that delays the commencement of breastfeeding by several hours [5]. This delay can disrupt the production of prolactin and impede the infant’s natural sucking reflex, leading to a decline in the mother’s confidence in her breastfeeding abilities [6]. Shockingly, an estimated 4,000 newborns and young children globally succumb daily due to not receiving breast milk [7]. Despite eventually transitioning to exclusive breastfeeding, many infants worldwide receive liquids other than maternal milk in the initial days after birth [8]. Particularly prevalent in Asian countries, pre-lacteal feeding exhibits high prevalence rates, with Bangladesh, Vietnam, India, and Nepal reporting rates of 77%, 73%, 49.9%, and 26.5%, respectively [9].

Initiating breastfeeding early, within the first hour of birth, is infrequent in Africa, representing a missed and significant opportunity for breastfeeding [2]. The prevalence of pre-lacteal feeding (PLF) in Burkina Faso and Egypt ranges widely from 11 to 58% [10]. Despite 52% of Ethiopian infants benefiting from breastfeeding within the first hour of birth, nearly three out of every ten children (27%) still receive pre-lacteal meals in the initial three days of life. The prevalence of this practice varies across different locations in Ethiopia, ranging from 10 to 72.5%, with the highest percentages reported in Dire Dawa, Harare, and Jimma, respectively [11].

The risk of mortality is heightened for infants who are not exclusively breastfed in the initial months, especially due to infections. Non-exclusive breastfeeding can increase an infant’s risk of succumbing to pneumonia and diarrhea in the 0 to 5-month age range by more than double [12]. Notably, pre-lacteal feeding has been associated with recurring illnesses, hindered growth, and an almost six-fold increased risk of mortality between the ages of 2 to 28 days compared to infants who receive early breastfeeding [13].

In a study conducted in West Gojam, Ethiopia, children who received pre-lacteal feeding were found to have a 1.8 times higher risk of stunting compared to those who did not engage in this feeding practice [14]. Similarly, research in the Ahmadabad area of Gujarat, India, showed that children who initiated pre-lacteal feeding (29.3%) had a higher prevalence of acute respiratory infection (ARI) compared to those who did not practice pre-lacteal feeding (16.3%) [15].

Numerous studies conducted across different regions globally consistently highlight the association of pre-lacteal feeding with various maternal healthcare factors (such as antenatal care attendance, mode of delivery, place of delivery, and delivery attendant), socio-demographic factors related to both child and mother (including maternal age, education, occupation, income, child age, sex, and birth order), maternal-related factors like breastfeeding difficulties, parity, and maternal illnesses, as well as cultural factors [16].

Despite Ethiopia’s efforts, such as the National Infant and Young Child Feeding (IYCF) Guideline discouraging pre-lacteal feeding practices, a range of harmful newborn feeding practices persist even after the implementation of infant and young child feeding guidelines. Although pre-lacteal feeding is widely practiced, there is limited research on its associated factors in Ethiopia, particularly in Dilla town, Southern Ethiopia. Therefore, the objective of this study is to assess the prevalence of pre-lacteal feeding and identify the associated factors among mothers with children under six months of age in Dilla town, Southern Ethiopia.

## Method

### Study area and period

The study was conducted in Dilla town, which serves as the administrative center of the Gedeo Zone in the Southern Nations, Nationalities, and People’s Region (SNNPR), Ethiopia, spanning from June 20 to August 20, 2022. Positioned 365 km away from Addis Ababa, the capital city of Ethiopia, Dilla is situated along the main road connecting Addis Ababa to Nairobi, Kenya. The town boasts essential healthcare facilities, including two health centers (Odaaya and Harroresa), one general hospital (Dilla University General Hospital), one primary hospital, 19 medium clinics, and five pharmacies. The total population of Dilla town is reported to be 97,516, with 49,733 females and 3,164 (3.19%) individuals under the age of one year. Among them, approximately 1,085 mothers have given birth within the last six months [17].

#### Study design

Community-based cross-sectional study design was used.

### Population

#### Source of population

All mothers or caregivers who had children of less than six months of age in Dilla town, Gedeo zone, Southern, Ethiopia.

### Study population

Mothers/care givers who had children of less than six months of age in the selected kebele of Dilla town was the study population. The sample unit was all selected house hold with mothers/care givers who had children of less than six months of age with in selected Kebele of Dilla town.

### Sample size determination

The sample size is determined by using a single population proportion formula. Considering the prevalence of pre-lacteal feeding practice of 12.6% obtained from the previous study conducted in Jinka Town South Ethiopia [18], assuming 95% confidence level, 5% margins of error, design effect of 2 and 10% non-response rate, as follows:

$${\rm{n}} = \frac{{{{({\rm{Z\alpha }}/2)}^2} \times {\rm{ p }}(1 - {\rm{p}})}}{{{{\rm{d}}^2}}}$$

Where Z = 95% CI (z = 1.96).

*P* = 12.6% from a study done in Jinka town.

d^2^ = marginal Error to be made, 0.05(5%).

$${\rm{n}} = \frac{{{{({\rm{Z\alpha }}/2)}^2} \times {\rm{ p }}(1 - {\rm{p}})}}{{{{\rm{d}}^2}}}$$

$$= \frac{{{{(1.96)}^2} {\rm X }\left( {0.126} \right)\left( {1 - 0.126} \right)}}{{{{(0.05)}^2}}}$$

$$= \frac{{3.8416{\rm{ X}}\,0.126{\rm{ }}\left( {0.874} \right)}}{{0.0025}}$$

$$\eqalign{ &= \frac{{0.423}}{{0.0025}}\cr&{\bf = 169}}$$

Then considering a design effect [2] and by adding 10% non-response rate *N* = 169 + 17 = **186**, the final sample size becomes 186*2 = **372**.

### Sampling technique and procedures

The study employed a two-stage sampling technique. In Dilla town, which comprises a total of 12 kebele, a subset of four kebele (Bareda, Haroke, Tena tabiya, and Bedecha) was randomly selected using a simple random sampling method. Subsequently, all households within these selected kebele with mothers having children aged less than 6 months were numbered. The total sample size was then proportionally allocated to each selected kebele, and a systematic sampling technique was applied to choose study participants based on their corresponding K value. During data collection, one eligible mother with a child aged less than 6 months was selected from each household unit. In cases where more than one potential respondent existed within a household, a simple random sampling method was employed to make the final selection.

### Eligibility criteria

The study included all mothers with children under six months in the selected kebele. However, mothers with contraindications for breastfeeding and those who were critically ill during the interview were excluded from the study.

### Study variables

The study investigated pre-lacteal feeding practice as the dependent variable, examining its relationship with various independent variables grouped into distinct categories. Socio-demographic factors encompassed maternal age, educational status, occupation, religion, marital status, child’s sex, birth spacing, economic status, and family size. Maternal health service utilization variables included antenatal care, place of delivery, and mode of delivery. Maternal-related factors consisted of parity, medical illness, and breastfeeding problems. Additionally, breastfeeding practices were explored through variables such as knowledge about the risks of pre-lacteal feeding, colostrum avoidance, breastfeeding initiation time, and perceived benefits of pre-lacteal feeding.

### Data collection tools and procedures

A structured interviewer administered questionnaire was used to collect the data which was adapted from relevant literatures and modified to local context. The questionnaire consisted of socio-demographic characteristics, maternal health care service utilization and obstetric characteristics, practice of breast feeding and colostrum feeding. The data was collected by the assigned data collectors after properly explaining the main purpose of the study. For mothers with more than 1 eligible child, the youngest was selected.

### Data quality control measures

Pretesting of the instrument was conducted before the actual data collection period on 5% (19 participants) mothers who live in Chichu district. The goal of the pre-testing was to guarantee that respondents could understand the questions and to rationally examine the phrasing, logic, and skip patterns of the questions. Amendments were made as a result of the pre-testing. Questionnaires were first prepared in English, then it was translated into Amharic and Gedeofa by language expert who has good ability of these languages, and then it was retranslated back into English to check consistency. Validity of the tool assured by senior experts of the study issue and its reliability was checked with cronbach’s alpha and it was 0.7 for maternal health care service utilization and obstetric characteristic related item.

### Data processing, analysis and presentation

The collected data was checked for completeness and consistencies. The data was coded, entered, cleaned and edited using SPSS version 23.0 software packages and during analysis, all explanatory variables which have significant association in bivariate analysis with a *P* value < 0.25 were entered into a multivariate logistic regression model to get AOR and those variables with 95% of CI and a *P* value of < 0.05 were considered as statistically significant with PLF. Multi-collinearity was checked to see the linear correlation between the independent variables by using variance inflation factor, and standard error. None of the variables yield inflation factor > 10, and standard error > 2 so they were not dropped from the multi – variable analysis. Hoshmer-Lemeshow test was found to be insignificant (*p* = 0.69) and Omnibus test was significant (0.000) which indicate that the model was fitted. Frequency tables, pie charts, and descriptive summaries were used to describe the study variables.

### Operational/standard definitions

#### Pre-lacteal feeding

is defined as giving fluid or semisolid before breast feeding to an infant during the first three days after birth. The mother was asked if she gave any drink other than breast milk to the child within the first three days of delivery. If she responded “yes” she considered as she had PLF practice and she responded ‘’no’’ she considered as she had no PLF practice [10].

#### Antenatal care utilization

having at least one visit of health institution for checkup purpose during the pregnancy of the index child.

#### Colostrum avoidance

includes; pumping and discarding colostrum during the first five days after birth [19].

#### Family size

everybody living permanently in the same house was counted as family member [16].

## Result

Three hundred seventy two mothers who had children less than 6 months of age were interviewed in this study, with a response rate of 100%. Most of the respondents were in the age group of 24–35. The majority of the respondents; 99 (26.6%) were house wives by occupation. Around half of the children, 198 (53.2%) were females (Table 1).

**Table 1:** Socio-demographic characteristics of mothers of children aged less than 6 months in Dilla Town, South Ethiopia, 2022 (*N* = 372)

Variables	Category	Frequency	Percentage
Type of respondent	Mother	361	97.0%
	Care giver	11	3.0%
Family size	<=3	91	24.5%
	> 3	281	75.5%
Age in years	15–24	125	33.6%
	24–35	211	56.7%
	> 35	36	9.7%
Marital status	Single	70	18.8%
	Married	282	75.8%
	Divorced	17	4.6%
	Widowed	3	0.8%
Educational status	unable to read and write	74	19.9%
	able to read and write	48	12.9%
	primary education	115	30.9%
	secondary education and above	135	36.3%
Religious group	Orthodox	138	37.1%
	Protestant	167	44.9%
	Muslim	50	13.4%
	Other	17	4.6%
Ethnic group	Oromo	31	8.3%
	Sidama	38	10.2%
	Gedeo	216	58.1%
	Gurage	42	11.3%
	Other	45	12.1%
Current occupation	private employee	49	13.2%
	civil servant	78	21.0%
	daily laborer	79	21.2%
	Merchant	19	5.1%
	Farmer	48	12.9%
	house wife	99	26.6%
Approximate family income per month	< 1000	126	33.9%
	1001–2000	98	26.3%
	2001–3000	44	11.8%
	> 3001	104	28.0%
Child’s age in month	< 1 month	162	43.5%
	1–3 month	103	27.7%
	3–5 month	59	15.9%
	5–6 month	48	12.9%
Child’s sex	Male	174	46.8%
	Female	198	53.2%

### Prevalence of pre-lacteal feeding practices

**Fig. 1:**
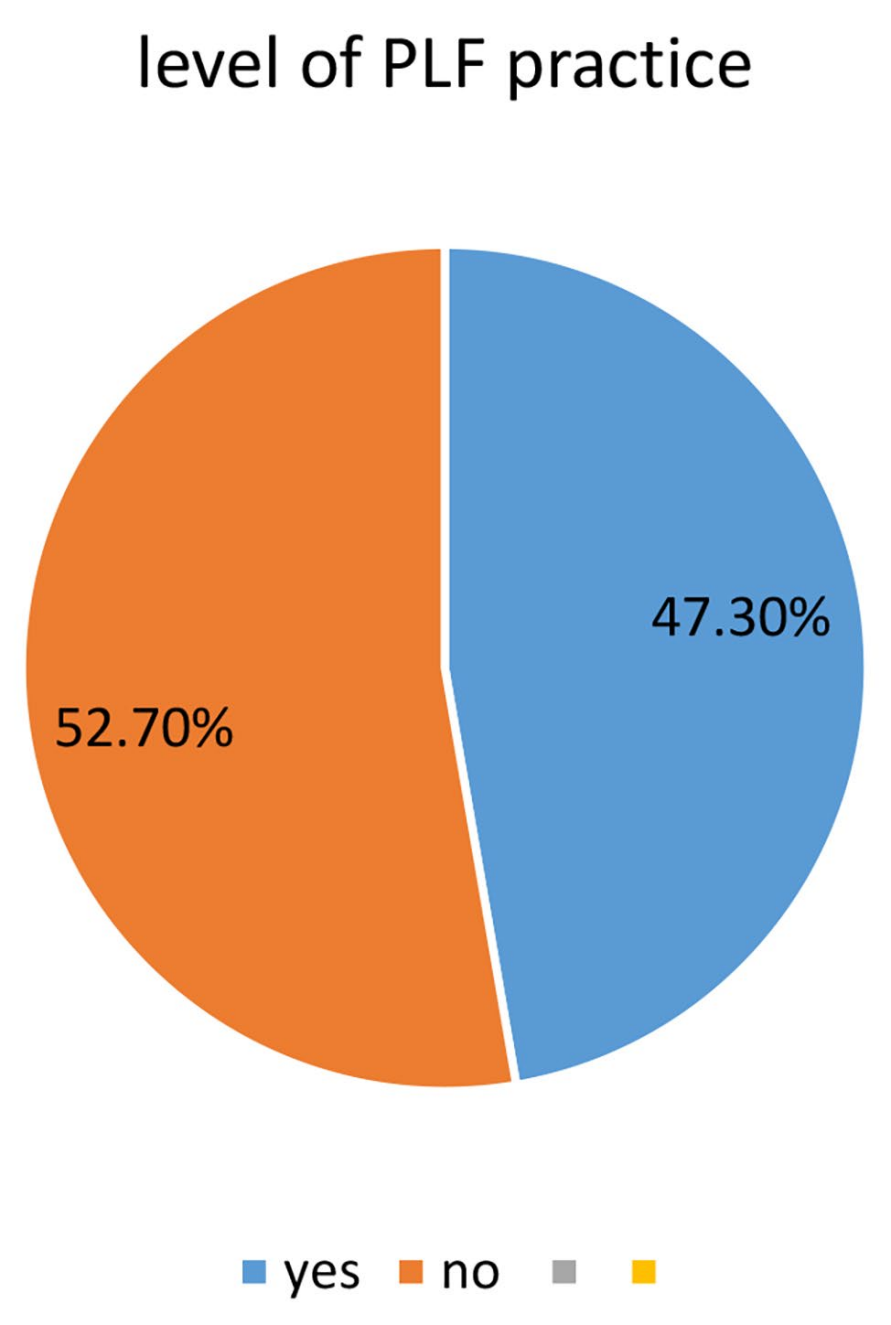
Prevalence of pre-lacteal feeding practices among children aged less than 6 months in Dilla Town, South Ethiopia, 2022 (*N* = 372)

The prevalence of pre-lacteal feeding practice in this study was 176(47.3%) with 95% CI [42.5– 52.2] (Fig. 1). This implies that 47.3% of study participants were reported that they have given pre-lacteal foods to their newborn in the first three days of birth. The most common type of pre-lacteal food were plain water (Table 2).

**Table 2:** Types of pre-lacteal food given for their children aged less than 6 months in Dilla Town, South Ethiopia, 2022 (*N* = 176)

Variable	Category	Frequency	Percentage
Plain water	Yes	49	27.9%
	No	127	72.1%
Glucose water	Yes	22	12.5%
	No	154	87.5%
Water with tenadam	Yes	37	21%
	No	139	79%
Butter	Yes	19	10.8%
	No	157	89.2%
Formula milk	Yes	28	15.9%
	No	148	84.1%
Other fluid	Yes	21	11.9%
	No	155	88.1%

### Decisions and reasons for pre-lacteal feeding practices

The majority of the respondents from those who practice pre-lacteal feeding (41.5%) gave pre lacteal feeding for their newborns with their own decision. Two hundred seventy-nine (75.0%) of respondents fed colostrum for their infants within the first five days after delivery and 93 (25.0%) of respondents avoided colostrum. The main reasons for colostrum avoidance were breast milk insufficiency 39(10.5%). Three hundred eighteen (85.5%) of mothers initiated breastfeeding within one hour (Table 3).

**Table 3:** Decisions and reasons for pre-lacteal feeding practices among mothers of children aged less than 6 months in Dilla Town, South Ethiopia, 2022 (*N* = 372)

Variables	Category	Frequency	percentage
My own decision	Yes	73	41.5%
	No	103	58.5%
Grand parents	Yes	40	22.6%
	No	136	77.4%
Traditional birth attendant	Yes	32	18.4%
	No	144	81.8%
My husband	Yes	30	17.1%
	No	146	82.9%
My friends	Yes	34	19.3%
	No	142	80.6%
Health professional	Yes	3	1.6%
	No	173	98.2%
Other bodies	Yes	23	12.9%
	No	153	86.9%
Did the infant get colostrum?	Yes	279	75.0%
	No	93	25.0%
Reason for colostrum avoidance	For the child growth	6	1.6%
	Brest did not have milk	39	10.5%
	Cause abdominal discomfort and diarrhea	26	7.0%
	Other	31	8.3%
Breast feeding initiation	Within 30 min − 1 h	318	85.5%
	1 h-1 day	54	14.5%

### Maternal health care service utilization and obstetric characteristics

Three hundred thirteen (84.1%) of mothers have used ANC services for their index infants. From those mothers who have used ANC services 228(61.3%) of them had more than four visits. About 294(79.0%) of respondents have got breastfeeding counseling. From those mothers who were counseled on breastfeeding 310(83.3%) were counseled on the benefits of breastfeeding (Table 4).

**Table 4:** Maternal health care service utilization and obstetric characteristics among mothers of children aged less than 6 months in Dilla Town, South Ethiopia, 2022 (*N* = 372)

Variables	Category	Frequency	Percentage
Did you attend ANC	Yes	313	84.1%
	No	59	15.9%
How many visits did you have?	1–4	228	61.3%
	> 4	85	22.8%
Counseled about breast feeding	Yes	294	79.0%
	No	19	5.1%
Counseling included its benefits?	Yes	310	83.3%
	No	3	8%
Parity	Primigravida	105	28.2%
	Multigravida	267	71.8%
Place of delivery	Gov’t health facility	283	76.1%
	Private clinic	85	22.8%
	At home	4	1.1%
Mode of delivery	CD	218	58.6%
	SVD	154	41.4%
Who assisted u during birth	Health professional	368	98.9%
	TBA	4	1.1%

#### Maternal information on PLF

**Fig. 2:**
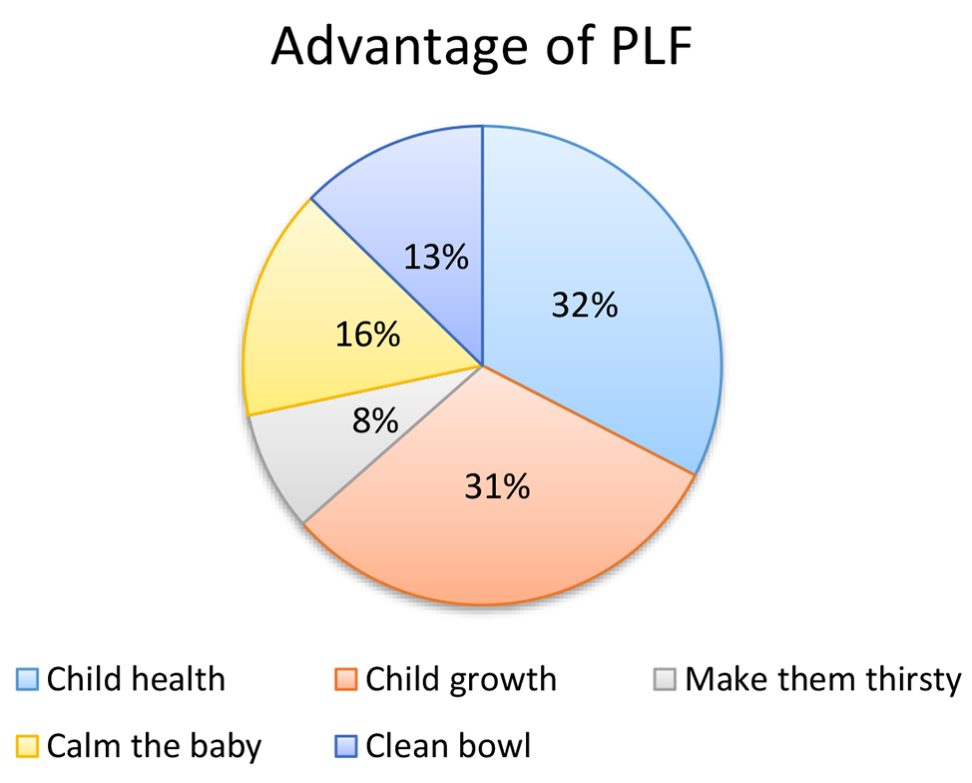
Maternal information on the advantage of PLF among mothers of children aged less than 6 months in Dilla Town, South Ethiopia, 2022 (*N* = 372)

**Fig. 3:**
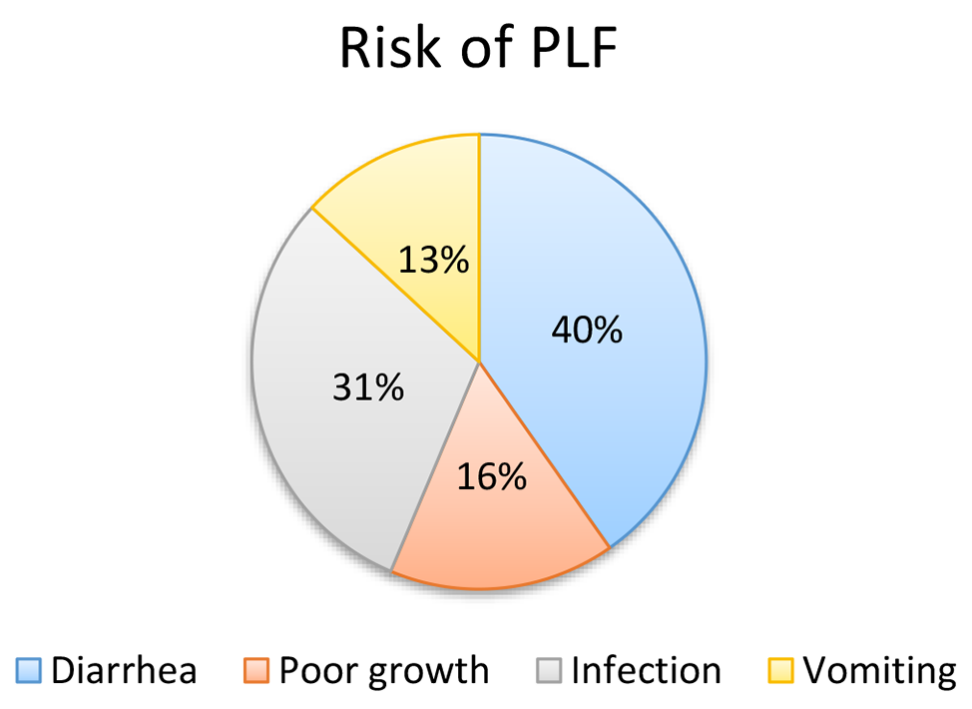
Maternal information on the risk of PLF among mothers of children aged less than 6 months in Dilla Town, South Ethiopia, 2022 (*N* = 372)

In this study, 307 (82.5%) mothers think that PLF has an advantage for their child and two hundred thirty-six (63.4%) of respondents stated that there is a risk associated with pre-lacteal feeding. Around 100 (32%) of the respondent believe that PLF is important for child growth (Fig. 2) and the majority of mothers which is 95(40%) reported that diarrhea is the major risk associated with PLF (Fig. 3).

### Factors associated with pre-lacteal feeding practices

On bivariable logistic regression educational status, current occupation, approximate family income, child sex, breast feeding counseling, benefits of breast feeding, place of delivery, mode of delivery and colostrum avoidance were associated with PLF practices. However, only educational status of the mother, colostrum avoidance and BF counseling were factors significantly associated with PLF practices in the multivariable logistic regression with *p* value of < 0.5 (Table 5).

**Table 5:** Factors associated with pre-lacteal feeding practice among mothers of children aged less than 6 months in Dilla Town, South Ethiopia, 2022

Variables	Category	PLF		COR(95% CI)	AOR(95% CI)
		Yes	No		
Educational status	unable to read and write	34	39	1.03(2.94,3.62)	3.68(1.01,5.84)**
	able to read and write	28	20	1.15(3.53,3.75)	
	primary education	52	63	0.9(0.01,0.24)	
	secondary education and above	62	74	1	1
Breast feeding counseling	Yes	102	192	1 5.29(2.25,3.30)	1 2.00(1.40,2.57)*
	No	19	0		
	Skip	55	4		
Colostrum avoidance	Yes	152	127	1	1
	No	24	69	0.23(0.13,0.41)	4.20(2.03,6.86)*

Note AOR = Adjusted Odds Ratio * = *P* < 0.05

COR = Crude Odds Ratio ** = *P* < 0.01

CI = Confidence Interval *** = *P* < 0.001

## Discussion

The prevalence of pre-lacteal feeding practice in Dilla town was found to be 47.3%, aligning closely with similar studies conducted in Eastern Ethiopia (45.4%), the Afar region (42.9%), Raya Kobo district (38.8%), Uganda (31.3%), and Karnakata, India (32.03%) [9, 20, 21]. These findings highlight a consistent occurrence of pre-lacteal feeding practices across various geographic locations, suggesting a shared challenge that extends beyond regional boundaries. Understanding the prevalence in Dilla town in comparison to other regions provides valuable insights into the broader context of early infant feeding practices and emphasizes the need for targeted interventions to address this widespread issue.

Nevertheless, the prevalence of pre-lacteal feeding practice in Dilla town, at 47.3%, was comparatively lower than that reported in other studies, such as in Vietnam (73.3%), Egypt (58%), and South Sudan (56%) [7, 22, 23]. The disparity in findings could be attributed to variations in the age of the children involved in the studies. Unlike the current investigation focused on mothers with children aged less than six months, the majority of the referenced studies examined mothers with children aged less than 24 months. This age difference may contribute to discrepancies, as mothers might encounter challenges recalling feeding practices from a more distant period, such as 24 months ago, potentially impacting the reported prevalence rates.

Furthermore, the results of this study revealed a higher prevalence of pre-lacteal feeding (47.3%) compared to the 2016 Ethiopian Demographic and Health Survey (DHS) report (7.9%) and studies conducted in East Wollega, West Ethiopia (6.7%) [18, 24, 25]. The variation between these findings could stem from differences in community attitudes towards pre-lacteal feeding across distinct ethnic groups. Additionally, socio-demographic disparities among the study participants may contribute to the observed inconsistency in pre-lacteal feeding practices, highlighting the influence of cultural and demographic factors on infant feeding behaviors within the studied population.

The findings of this study underscored the significance of maternal education in influencing pre-lacteal feeding (PLF) practices. Mothers who lacked formal education, unable to read or write, were found to be 3.68 times more likely to engage in pre-lacteal feeding compared to mothers with secondary and higher levels of education. This aligns with similar conclusions drawn from studies conducted in Nepal, Debre Markos Town, and Mettu District [26–28]. The association between lower maternal education and a higher likelihood of pre-lacteal feeding could be attributed to the enhanced understanding among educated mothers regarding the importance of proper breastfeeding, which may act as a deterrent against initiating pre-lacteal feeds. Conversely, mothers with limited education may be more susceptible to external influences from traditional birth attendants and grandparents, potentially contributing to the practice of pre-lacteal feeding.

The study revealed that mothers who did not initiate breastfeeding with colostrum for their index infants within the first five days were approximately four times more likely to engage in pre-lacteal feeding compared to mothers who did provide colostrum. This finding is consistent with research conducted in various locations, including Axum Town, Mettu District, Motta Town, and North Eastern Ethiopia [5, 28]. The connection between avoiding colostrum and a higher likelihood of pre-lacteal feeding may be attributed to the decline in the infant’s suckling activity, leading to reduced breast stimulation and subsequently decreased maternal milk secretion. This, in turn, could compel the mother to introduce other foods to the infant. The perception of colostrum as unclean or harmful to the infant’s health may contribute to this practice among mothers.

According to the findings of this study, mothers who did not receive breastfeeding counseling were twice as likely as their counterparts to practice pre-lacteal feeding (PLF). This aligns with similar observations in Vietnam and South Sudan [7, 23]. The association between lack of breastfeeding counseling and a higher likelihood of pre-lacteal feeding could be explained by counseling serving as a crucial strategy to assist mothers in modifying their behaviors and refraining from engaging in pre-lacteal feeding during pregnancy. Prenatal breastfeeding counseling has the potential to enhance a mother’s understanding of optimal breastfeeding practices, thereby potentially reducing the incidence of pre-lacteal feeding practices.

### Limitation of the study

This study’s strength lies the comprehensive two-stage sampling technique and inclusion of various socio-demographic and healthcare variables enhance the depth of understanding regarding factors associated with pre-lacteal feeding practices. The comparison with national and global data adds a broader perspective. However, limitations include the cross-sectional design, introducing constraints in establishing causation or temporal trends, and the reliance on mothers’ recall, susceptible to bias. Social desirability bias and potential confounders may affect the accuracy of reported data. Generalizability is limited to Dilla town, and the study’s findings may not entirely represent other regions or settings. Nonetheless, this research contributes valuable insights into local practices and factors influencing pre-lacteal feeding.

## Conclusion

The prevalence of pre-lacteal feeding practices among mothers with children aged less than 6 months in Dilla town was identified to be higher than the national prevalence. The independent factors significantly associated with pre-lacteal feeding practices included maternal inability to read and write, colostrum avoidance, and the absence of breastfeeding counseling. These findings highlight specific areas of concern within the community and emphasize the importance of targeted interventions to address factors influencing pre-lacteal feeding practices in this population.

## Data Availability

The datasets generated and /or analyzed during the current study are not publicly available due to preserving participant anonymity but are available from the corresponding author on reasonable request (Anteneh Gashaw, antenehgashaw77@gmail.com ).

## Abbreviations

PLF: Pre-Lacteal Feeding
SNNPR: South Nation Nationalities and Peoples Region
CI: Confidence interval
COR: Crude odds ratio
AOR: Adjusted Odds Ratio
